# Pedometer use and self-determined motivation for walking in a cardiac telerehabilitation program: a qualitative study

**DOI:** 10.1186/s13102-016-0048-7

**Published:** 2016-08-18

**Authors:** Charlotte Brun Thorup, Mette Grønkjær, Helle Spindler, Jan Jesper Andreasen, John Hansen, Birthe Irene Dinesen, Gitte Nielsen, Erik Elgaard Sørensen

**Affiliations:** 1 Department of Cardiothoracic Surgery, Aalborg University Hospital, Hobrovej 18-22, DK-9000 Aalborg, Denmark; 2 Clinical Nursing Research Unit, Aalborg University Hospital, Søndre Skovvej 15, DK-9000 Aalborg, Denmark; 3 Department of Psychology and Behavioural Science, Aarhus University, Bartholins Allé 9, DK-8000 Aarhus C, Denmark; 4 Department of Clinical Medicine, Aalborg University, Søndre Skovvej 15, DK-9000 Aalborg, Denmark; 5 Laboratory for Cardio technology, Medical Informatics Group, Department of Health Science and Technology, Faculty of Medicine, Aalborg University, Frederik Bajers Vej 7D, DK-9220 Aalborg, Denmark; 6 Laboratory of Assistive Technologies - Telehealth and Telerehabilitation, SMI, Department of Health Science and Technology, Faculty of Medicine, Aalborg University, Aalborg, Denmark; 7 Department of Cardiology, Vendsyssel Hospital, Bispensgade 37, DK-9800 Hjoerring, Denmark; 8 Department of Health Science and Technology, Faculty of Medicine, Aalborg University, Frederik Bajers Vej 7D, DK-9220 Aalborg, Denmark

**Keywords:** Pedometer, Physical activity, Telemedicine, Cardiac telerehabilitation

## Abstract

**Background:**

Exercise-based cardiac rehabilitation reduces morbidity and mortality. Walking is a convenient activity suitable for people with cardiac disease. Pedometers count steps, measure walking activity and motivate people to increase physical activity. In this study, patients participating in cardiac telerehabilitation were provided with a pedometer to support motivation for physical activity with the purpose of exploring pedometer use and self-determined motivation for walking experienced by patients and health professionals during a cardiac telerehabilitation program.

**Methods:**

A qualitative research design consisting of observations, individual interviews and patient documents made the basis for a content analysis. Data was analysed deductively using Self Determination Theory as a frame for analysis and discussion, focusing on the psychological needs of *autonomy, competence* and *relatedness*. Twelve cardiac patients, 11 health professionals, 6 physiotherapists and 5 registered nurses were included.

**Results:**

The pedometer offered independence from standardised rehabilitation since the pedometer supported tailoring, individualised walking activity based on the patient’s choice. This led to an increased *autonomy*. The patients felt consciously aware of health benefits of walking, and the pedometer provided feedback on walking activity leading to an increased *competence* to achieve goals for steps. Finally, the pedometer supported *relatedness* with others. The health professionals’ surveillance of patients’ steps, made the patients feel observed, yet supported, furthermore, their next of kin appeared to be supportive as walking partners.

**Conclusion:**

Cardiac patients’ motivation for walking was evident due to pedometer use. Even though not all aspects of motivation were autonomous and self determined, the patients felt motivated for walking. The visible steps and continuous monitoring of own walking activity made it possible for each individual patient to choose their desired kind of activity and perform ongoing adjustments of walking activity. The immediate feedback on step activity and the expectations of health benefits resulted in motivation for walking. Finally, pedometer supported walking made surveillance possible, giving the patients a feeling of being looked after and supported.

**Trial registration:**

Current study is a part of The Teledi@log project.

## Background

Cardiac rehabilitation (CR) decreases morbidity and mortality [1, 2]. Home-based cardiac rehabilitation such as cardiac telerehabilitation (CTR) appears safe and effective in improving physiological and psychological health outcomes compared to hospital based or healthcare centre based CR [3–5]. Telerehabilitation is the application of telemedicine and telecommunication technology for supporting rehabilitation services [6, 7]. It is potentially more cost-effective for patients living far from their local health centre or hospital [8], and may result in longer lasting maintenance of physical activity levels compared to hospital-based rehabilitation [8, 9]. In particular, exercise-based CR reduces morbidity and all cause and cardiovascular diseases mortality [10–12]. Furthermore, the risk of re-hospitalisation [13] and re-infraction is decreased [14]. In addition, exercise based CR reduces progression of the disease, functional limitation [15], levels of depression, anxiety, hostility and total psychological stress [9, 12–14, 16, 17]. Walking is a simple and convenient physical activity suitable for people with cardiac disease [18, 19] and provides a flexible and alternative form of activity for those unable to access hospital-based programs [18]. Additionally, it has a crucial function in the resumption of work and daily life [18]. Activity monitors, such as pedometers, are designed to count steps and measure walking activity. Using pedometers by setting activity goals motivates patients to increase physical activity [20, 21] thereby improving physiological and psychological health outcomes for cardiac patients [22, 23]. Furthermore, pedometers are useful in observing levels of activity and indicate adherence to activity programs [21, 23–25] up to 12 months after walking intervention [26].

Motivation is essential for sustained behavioural change [27, 28], and interventions based on behaviour change theories seem more effective than those lacking a theoretical basis [3]*.* In relation to physical activity, Self Determination Theory (SDT) provides a theoretical framework for long-term motivation for behaviour change. Thus, researchers have begun to implement physical activity recommendations grounded in SDT [29–32]. To qualify aspects of motivation for walking among patients in a CTR program, this study used SDT as a frame for analysis and discussion. According to SDT, there are three psychological needs that shape behaviour and motivation: *autonomy* (choice, volition and freedom), *competence* (confident in being able to perform the behaviour and achieve a desired goal) and *relatedness* to other humans (positive, warm relations) [27–34]. Fulfilment of the three psychological needs seems to form the basis for autonomous motivation [27–34]. Motivation lies along a continuum with varying degrees of self-determination [30–34] including both intrinsic and extrinsic components. Intrinsic motivation involves motivation derived from pleasure and satisfaction of performing the behaviour itself. Extrinsic motivation involves a decrease in autonomous regulations while behaviour becomes more controlled. If the behaviour is caused by expectations from others (controlled), motivation becomes less autonomous and more extrinsic. Motivation at the most intrinsic end of the continuum seems to form more sustainable behaviour. By internalisation of for instance extrinsic values motivation may become more autonomous and self determined as the person values the behaviour as important for their own identity. The most complete form of internalisation is called integration [31–35].

This paper presents findings from a sub-study of a randomised controlled CTR trial called Teledi@log [36] in which an intervention group of cardiac patients were provided with telerehabilitation technology for a 3 month monitoring period. In Teledi@log motivational aspects were inspired by SDT. The majority of previous pedometer studies have focused on increase or decrease in step activity [22, 37–39]. However, there is a lack of knowledge on patient experiences of pedometer use as motivation for physical activity. Furthermore, it seems SDT has the potential to explain yet unrevealed aspect of motivation in relation to pedometers based on walking activity for cardiac patients in a telerehabilitation program. Thus, the aim for this study was to explore pedometer use and self-determined motivation for walking during a cardiac telerehabilitation program from patients’ and health professionals’ experiences.

## Methods

### Teledi@log

In Teledi@log the telerehabilitation technology consisted of a Fitbit Zip pedometer [40], a weight scale, a sphygmomanometer and a tablet computer, which contained a tailored personal health record (PHR) for health information and communication between the patients and health professionals. After discharge from hospital, the patients themselves measured blood pressure, pulse and weight twice a week and steps daily for a three-month period. This measurement frequency was considered as basic measurement for all participants in Teledi@log, and none of the participants in this sub-study were prescribed by the hospital doctors, to make any additional measurements. Step data were continually visible on the step counter’s display, and each day at midnight all data (incl. step data) were downloaded into the patient’s PHR. All patients were assigned a personal rehabilitation nurse who created rehabilitation plans tailored to the patients’ needs in collaboration with the patients. Plans for physical activity and walking were made in collaboration with a rehabilitation physiotherapist. The individual rehabilitation plans were displayed in the PHR, which both the health professionals and the patients had access to and used for communication. All patients had personal goals for daily steps and were provided with access to records of their own walking activity. In the PHR, the rehabilitation nurses gave feedback to the patients regarding rehabilitation and walking activities, e.g. by writing encouraging notes. In addition to access to health information, the pedometer was the only telerehabilitation technology the patient retained after 3 months, and the monitoring of steps continued for 12 months. The Teledi@log project that has the trial Registration: ClinicalTrial.gov: NCT01752192.

The methodology for this study was inspired by ethnography, thus consisted of participant observations, individual interviews and documents from the patients’ PHR [41–46]. Patients were observed twice focusing on their usage of the pedometers and they were interviewed to investigate how they experienced their use of the pedometer during and after the monitoring period. Health professionals were interviewed to discover how they experienced using a pedometer as a motivation tool for patients walking activity [45, 46]. The documents consisted of written digitalised communication between the patients and health professionals derived from the PHR [43, 44].

### Participants and procedures

The participants comprised 12 patients from the Teledi@log trial, 11 health professionals, 6 physiotherapists and 5 registered nurses (RN) responsible for the CTR in the Teledi@log trial. All participants were approached by the first author, and signed informed consent after agreeing to participate in this sub-study. The nature of the studies was explained in writing and verbally to the participants, and all participants were provided with anonymity and confidentiality, thus all names of participants used in this paper are fictional.

#### Patients

From June until September 2013, patients were consecutively selected from the Teledi@log trial. The inclusion criteria were 18 years of age or more, acute coronary syndrome (ACS), heart failure or coronary artery bypass surgery or valve surgery. The exclusion criteria were pregnancy, breastfeeding or an inability to speak Danish.

Participant observations were performed twice in the patient’s home [43, 44]. The first observation period took place 2 weeks after discharge, and the second observation period occurred 3 months later. The observations focused on the patients’ mnemonic strategies for using the pedometer, the pedometer’s placement on the body, and the patient’s ability to view walking activity on the pedometers display and in the PHR. The time span between the two observation periods made it possible to focus on the patients’ achieved routines for pedometer use at the second observation. The observations lasted from 30 to 45 min. They were digitally recorded by the first-author immediately after each observation, and transcribed verbatim before conducting the patient interviews.

The interviews took place in the patient’s home one month after the second observation period, as this time frame was considered relevant for investigating the patients’ experience of using the pedometer during and after the monitoring period [43, 45]. The interviews were based on a semi-structured guide inspired by SDT [33, 35] (Appendix 1), events from the observations and notes from the PHR [43, 44]. The interviews lasted from 45 to 75 min. The analysis of the transcribed interviews demonstrated that a satisfactory level of saturation was reached [47], and no further interviews were conducted.

#### Health professionals

Eleven registered nurses (RN) and physiotherapists participated. They were purposefully selected as the most experienced in promoting motivation using a pedometer because they had been a part of the Teledi@log research right from the beginning, and totalled all health professionals participating in the Teledi@log trial. They were recruited from a University Hospital, a Regional Hospital and four Healthcare Centres. The interviews focused on health professionals’ experiences of using a pedometer as a motivational tool for activity. The interviews were based on a semi-structured guide inspired by SDT [33, 45] (Appendix 2) and notes from the PHR [43, 44]. The interviews lasted approximately 20–35 min and were digitally recorded and transcribed verbatim.

#### Documents

The tailored PHR served as an interactive platform with updated information about cardiac disease, prevention of disease progression and the patient’s weight, blood pressure and steps. The patients’ individual rehabilitation plans were displayed in the PHR, which both the health professionals and the patients had access to and used for communication. Notes from the PHR were used as data.

### Data analysis

Data were analyzed using deductive content analysis [42] with SDT as a frame for analysis. Deductive content analysis is useful when the intention is to describe the phenomenon in a conceptual form. Deductive content analysis is appropriate when the structure of analysis is made on the basis of previous knowledge or theory [42, 46]. As such, a deductive approach was used as the aim was to reveal new aspects of an experience of pedometer use within the theoretical framework of SDT.

The units of analysis (data) were transcribed text from observations and interviews and notes from the PHR (documents) [41, 42]. Trustworthiness was supported by this broad range of data used, as multiple aspects on experience of pedometer use may emerge [48]. All data were organized using the software package Nvivo10 (Nvivo qualitative data analysis software; QSR International Pty Ltd. Version 10, 2014). After an in-depth reading of the data ‘units of meaning’ were identified and coded. Codes were grouped in sub-themes, and then abstracted into themes inspired by the three psychological needs presented in the SDT: autonomy, competence and relatedness. In addition to the deductive approach, data was sought for spontaneous issues raised by the participants [42, 45]. The analysis resulted in three themes each with two sub-themes. Parts of the content analysis, with authentic citations, are presented in Appendix 3. To support trustworthiness of the research, all findings were discussed continuously between the authors [42].

## Results

The included patients were 8 men and 4 women with a median age of 62 years (range: 36–85 years). One female died during the study period, leaving 11 patients available for interviews. Five nurses and 6 physiotherapists were included. All participants and their characteristics are displayed in Tables 1 and 2.

**Table 1:** Treatment, age and gender of the included patients

ID	Sex	Treatment
1	Male	Surgery
2	Male	Surgery
3	Female	Surgery
4	Female	Medical
5	Male	Medical
6	Female	Medical
7	Male	Surgery
8	Male	Medical
9	Female	Medical (deceased)
10	Male	Medical
11	Male	Surgery
12	Male	Surgery
Mean and range age		62 (36 – 85)

**Table 2:** Workplace and gender of the included health professionals

	Physiotherapists	RN
University Hospital	1 (Male)	
Regional Hospital	1 (Female)	1 (Female)
Healthcare Centre 1	1 (Female)	1 (Females)
Healthcare Centre 2	1 (Male)	1 (Female)
Healthcare Centre 3	1 (Female)	1 (Female)
Healthcare Centre 4	1 (Female)	1 (Female)

The analysis revealed three themes and six subthemes according to each of the three psychological needs from SDT. The first theme ‘Autonomy as independence from standardised rehabilitation’ had the following two subthemes: *Individual choice and decision for walking activity* and *Tailoring walking activity*. The second theme: ‘Competence as conscious awareness of walking activity’ had the following two subthemes: *Feedback on walking activity* and *Knowledge leading to awareness of walking*. The third was: ‘Relatedness as interaction with others in relation to walking activity’, with the following two subthemes: *Feelings of being under surveillance, yet supported* and *Support from the next of kin*. Themes and subthemes are displayed in Table 3. In the following, themes and subthemes are expounded and supported with quotations from observations (field note ID), interviews (ID) and notes from the PHR (PHRpatientID or RN/Physiotherapist Healthcare Centre).

**Table 3:** Themes and subthemes from the content analysis

Psychological needs from SDT	Themes from analysis	Subthemes from analysis
Autonomy	Independence from standardised rehabilitation	Individual choice and decision for walking activity
		Tailoring walking activity
Competence	Conscious awareness of walking activity	Feedback on walking activity
		Knowledge leading to awareness of walking
Relatedness	Interaction with others in relation to walking activity	Feelings of being under surveillance, yet supported.
		Support from next of kin

### Autonomy as independence from standardised rehabilitation

#### Individual choice and decision for walking activity

The patients gained insight into their own activity because their steps became visible, and they felt an opportunity to decide for themselves what kind of activity they wanted to perform. As such, goals for steps became flexible, and for some, the activity was incorporated into their daily living: “*When the lawn needs mowing, then you feel motivated, not to be active, but to make the lawn look good” (ID8)*. One patient lost the pedometer and felt no need for it anymore: “*I don’t need the pedometer anymore. I now know how many steps the normal working day provides, or my favourite walking trip” (ID5)*. It seemed that the pedometer provided independence, also after termination of use, enabling the patients to choose exercise by themselves without attending traditional rehabilitation. The patients felt capable of exercising on their own guided by the PHR and the pedometer: *“The alternative was to exercise at the Healthcare centre, but I am not driving all that way. You could just take a walk in the nature. It’s basically the pedometer that supports my exercise” (ID11),* which was supported by this field note: *“In the living room there was a rowing machine, and during the first observation the patient expressed a wish for a more detailed personal plan for exercises improving strength beside her pedometer goals. She explained that this was because she wanted to exercise on her own” (Field note ID3).* On request, the physiotherapist made an individual exercise program in this patient’s PHR. Thus, the pedometer made the patients consider their step goals as flexible rather than fixed. Because the visible steps disclosed the individual activity, this led to increased independence from standardised rehabilitation leaving space for individual choice and decision.

#### Tailoring walking activity

The patients used the pedometers to monitor steps across different activities. They chose activities suitable for their lifestyle and developed strategies to achieve their step goals through these activities. This made them independent of standardised walking programs, i.e. some walked to the grocery store instead of driving. Furthermore, they modified their activity by going for a walk if their amount of steps had not reached a satisfactory level, at a time suitable for their daily life. As such, the pedometer became a tool that made it possible for them to tailor activity individually: One patient expressed, “*When you have a pedometer, you look at it, how many steps have I walked now? Then we’ve gone for an evening walk. If you can’t see the results of what you do, you have no opportunity to adjust” (ID10).* In other words: “*Before [the pedometer] I wasn’t given any marker on how many steps to walk a day. I was just told to walk; now I walk longer distances about 7000 steps 8000 steps on one trip” (ID2).* Similarly, the health professionals expressed that monitoring steps made it possible to assist in tailoring each patient’s individual training program. A physiotherapist explained, *“In worst case scenarios they only walked 2000 steps in a day. You have to be aware of their starting point when you plan their individual activity level” (Healthcare Centre 4).* The new possibilities for tailoring activity plans were also expressed in the PHRs. The RN wrote, *“John experiences leg pain when walking. We agreed to measure how far he can walk (numbers of steps). After that we will determine goals for daily steps” (PHRpatientID1).* As stated above, monitoring the patient’s steps made it possible to adjust and tailor walking activity for each individual patient. As such, individual strategies for walking activity became obtainable.

### Competence as conscious awareness of walking activity

#### Feedback on walking activity

The visibility of steps on the pedometer and in the PHR provided the patients with immediate feedback on the amount of steps walked. The patients wanted to achieve step goals because it gave them satisfaction. A patient expressed, *“It’s nice to see that I did actually walk many steps today.” (ID1).* A nurse expressed that consciousness about patients’ walking activity increased due to the visibility of step activity: *“Previously it seemed blurred, whereas now, with this [the pedometer], it is easier to keep track on their activity” (Healthcare Centre3).* Likewise, the notes in the PHR revealed that patients were consciously aware of walking and became dissatisfied if they forgot the pedometer: “*Unfortunately I forgot the pedometer this morning, and I went for a long walk, which unfortunately didn’t get registered” (ID2).* Another patient wrote in the PHR: *“Hi Mette [nurse at the hospital], the pedometer is really motivating. I wore it at the gym, I went there with my wife, and it gave me 2 – 3.000 steps at the cross trainer” (PHRpatientID12).* Visibility, as the immediate feedback on walking activity, supported the patients’ competence in walking activity because it made both the patients and health professional consciously aware of patients achieved steps.

#### Knowledge leading to awareness of walking

Health professionals and patients jointly expressed that the awareness of health benefits from walking made patients walk with intent. Walking became more than an everyday activity of getting around, it became a conscious activity supporting health. A nurse said: *Some of the patients don’t consider walking as a health related issue; they just consider walking as an act to get from one place to another. The pedometer changed that” (Healthcare Centre 1).* This was supported by a patient: “*It is all about health. I try to keep as healthy as possible, and it appears that exercise makes a difference” (ID10).* Another said, “*Walking is my work. I want to be in a good shape, because it’s good for me” (ID2).* Some patients deliberately tried to increase their awareness of their walking by placing the pedometer visibly on the body: “*A patient had the pedometer placed visibly at the shirt, expressing that for him this was an appropriate place because it reminded him of walking” (Field note ID11)*. As such, the pedometer increased their focus on health benefits of walking activity, and made patients aware of walking. This knowledge supported the feeling of being competent in performing the activity.

### Relatedness as interaction with others in relation to walking activity

#### Feelings of being under surveillance, yet supported

The patients seemed aware of their step activity being monitored by health professionals, and they did not want to “lose face” or to disappoint the health professionals due to inactivity. They felt motivated to verify their step activity: *“You lose face if the pedometer shows too few steps. I mean, you lose face if you don’t do what they [the health professionals] told you to do” (ID4).* In the PHR a patient evaluated the surveillance: “*It’s a safe feeling, that the nurse follows your rehabilitation status in the PHR. I haven’t reach my goals yet, but I am determined that I will” (PHR ID9).* They wanted to explain their activity to the health professionals, as seen in a PHR: “*Hallo Mette [nurse at the hospital]. As you can see, I don’t walk much. I am extremely affected by the new drug. Immediately after intake, my pulse and blood pressure drops and I need to lie down” (PHRpatientID4).* This illustrated that the patients were motivated to walk more because they were under surveillance by the health professionals. In addition, the health professionals used the PHR to support the patients’ motivation by giving feedback on their activity. A physiotherapist expressed: *“For the patients, it´s the immediate result each evening. It’s like a close surveillance of activity, like a: ‘well done today, Peter’” (Healthcare Centre2),* and, in a patient’s PHR a nurse wrote: *“Hi Hans. I can see that you have been really active, that’s good☺. Enjoy the lovely weather today, maybe you feel like a long walk on the beach?”(PHR patientID5).* As stated above, the relation between patients and health professionals involved surveillance in a supportive way. The patients strived at achieving and documenting sufficient walking activity and the health professionals aimed at supporting motivation by positive comments on walking activity.

#### Support from next of kin

Patients expressed that relatives and friends may be supportive for walking motivation. A patient walked with her children and expressed: *“They helped me to get started. And when you have the pedometer, then you look at it, ‘how many steps have I been walking?’, then we have been out for an evening walk” (ID3)*, and another walked with a friend: *“I just call my friend and ask her; ‘don’t you need some fresh air?’ It’s like; ‘two for the price of one’ because then we talk and talk, and suddenly, without noticing, we have been walking a long trip (ID6)”*. A physiotherapist had noticed a friendly competition between a man and his wife*: “His wife bought a pedometer herself, they compared, and talked about how many steps they had reached. I think that it was motivating for them, to see the spouse feeling good” (Healthcare Centre3).* As such, relatives and friends seem to support the patients’ motivation for walking due to friendly competition and a wish to walk with others.

## Discussion

The present study revealed that the pedometer offered independence from standardised rehabilitation. Step goals became flexible and the pedometer provided opportunities to tailor activities in respect to daily living. This led to an increased independence and *autonomy*. In SDT, intrinsic motivation forms the basis for autonomous or self determined behaviour [33]. In addition, tailoring, i.e. the possibility to make an individualised choice supports the patient’s feeling of being able to perform the activity, in turn supporting motivation for behavioural changes [49, 50]. This study revealed that visible steps gave the patients a tool to choose and decide their own activity taking their own interests and values into account. Studies reporting barriers to motivation for lifestyle changes point out that tailoring of interventions is important for sustained behavioural changes [3–5, 50]. Thus, independence formed by choice, decision and tailoring may be viewed as integrated motivation at the most intrinsic and autonomous end of the motivation continuum. The possibility for sustained motivation for walking was present.

The pedometer provided a conscious awareness of walking activity. The immediate feedback on step activity supported the awareness of walking activity for both patients and health professionals leading to an increased *competence* to achieve goals for steps. Furthermore, the increased awareness of health benefits of walking made walking activity an informed choice. According to SDT, competence is formed by the person’s beliefs of being able to carry out the desired behaviour change [22, 25, 37]. Furthermore, setting realistic goals shapes the person’s feeling of confidence in performing behavioural changes [22]. In this study, the pedometer became a tool to set clear and individual goals for walking and to make the patients aware of their own level of activity. According to Bratava et al. [20], pedometer users who were given daily step goals significantly increased their physical activity, whereas pedometer users without step goals did not increase their physical activity. The present study showed that patients felt motivated to reach their step goals because they expected health benefits and they became aware of walking as a healthy activity. From a SDT perspective, this may be viewed as extrinsic motivation because the motivation is not the act itself (the walking) but the expected achievements of the act (the health benefits), yet the motivation seems integrated because it stands well-internalised [31]. Studies on sustained motivation for behavioural changes fail to show long time effects when motivation is extrinsic, but short term effect are evident [33, 35]. As such, competence as awareness of walking cannot be seen as entirely intrinsic [31–35] and must be placed closer to the less self determined end of the continuum.

The pedometer supported interaction with others in relation to walking activity. *Relatedness* was expressed both as surveillance and support, as the patients felt observed, yet supported by health professionals and helped by their next of kin. Surveillance put pressure on the patients supporting their attempt to achieve and document sufficient walking activity, despite that, the health professionals maintained a supportive role. The surveillance increased the attempt to fulfil goals for daily steps thereby motivating patients to walk. In addition, the health professionals gave positive feedback by writing supportive comments in the PHR. According to SDT this can be seen as an integrated extrinsic motivation that support the motivation for walking, despite the fact that SDT consider surveillance and feedback (e.g. standardised text messages) as failing to provide long term motivational changes, because the behaviour stops as the surveillance and feedback stops [33, 35]. Furthermore, motivation based on ‘not losing face’ is extrinsic as it is not the act itself that motivates. Regardless, patients felt motivated, supported and safe while being observed. This is in line with other telerehabilitation studies in which surveillance and being observed is found to support motivation for behavioural changes [3, 4]. In SDT, relatedness occurs if significant others demonstrate understanding and involvement and significant others may be friends, family but also health professionals [35]. The next of kin also supported the patients walking activity by friendly competitions and by being a ‘walking partner’. In our study, the next of kin showed involvement by friendly competition and by going for walks with the patients. This seems to support the feeling of understanding and involvement and thereby supporting intrinsic motivation. Studies have shown that pedometers may improve walking activity and that home-based rehabilitation exercise may have longer-lasting effects than hospital-based rehabilitation because it seems like more of a lifestyle change than treatment [8, 20]. Furthermore, telerehabilitation technology has the potential to overcome barriers in access to CR and to reach a wide segment of the population [3–5, 8]. However, it is outside the scope of this study to determine whether walking activity or participating CR was increased.

### Strengths and limitations

The participant observations and patient interviews were conducted in private settings that provided a relaxed and comfortable atmosphere for the participants. The in-depth reading and analysis was undertaken only by the first author leading to a risk of mis-interpretation. To avoid this risk of bias an ongoing discussion of the analysis and interpretation was made with the co-authors. De-contextualisation of the text might appear when using Nvivo10, but the critical reading and discussion between the authors was performed to avoid this. Even though the pedometer Fitbit Zip seems to be valid in measuring free-living physical activity [51, 52] it has shown high step error at slow walking speed [53], and the walking speed seems to be slow in older people [54], and in patients with cardiac disease [55–57]. None of the patients addressed any spontaneous concerns or mistrust regarding the reliability of the pedometer.

The researchers were part of the Teledi@log project, which might provide blind spots in the analysis and interpretation because familiarity to Teledi@log may lead to truisms. On the other hand, this familiarity may have provided a deeper understanding of the research topic.

## Conclusion

Pedometers offered independence from standardised rehabilitation and made it possible for the patients to tailor, choose and decide time and place for walking activity. Furthermore, the pedometer provided a conscious awareness of walking activity due to the immediate feedback on step activity for both patients and health professionals. Besides that, patients felt an increased awareness of health benefits of walking and they strived to achieve goals for steps. Finally, the pedometer supported interaction with others in relation to walking activity. The patients felt under surveillance and supported by health professionals and they felt helped by their next of kin. Even though not all aspects of motivation were entirely intrinsic they seemed integrated. Thus, the patients felt motivated and engaged in walking activity.

## Abbreviations

ACS, acute coronary syndrome; CR, cardiac rehabilitation; CTR, cardiac tele-rehabilitation; PHR, personal health record; RN, registered nurse; SDT, self determination theory

## Appendix 1

**Table 4:** Interview guide patients

*Opening question*	
Who, time and place	Would you please introduce yourself briefly by telling your name, age and your illness?
*Research questions*	*Interview questions*
Covering the following areas • Importance of physical activity • Autonomy • Competence • Relatedness • The use of the step counter	What does physical activity mean to you (what is important)? What do you expect from being physical active? In relation to physical activity, what do you expect to achieve during the next yare? If others should describe your way of being physical active, what would they say? What are your advantages in relation to physical activity? What is most challenging for you, in relation to physical activity? In relation to physical activity, what would you appreciate to learn more about? What behaviour you like to changes in relation to physical activity? What persons’ do influence you to change your level of activity (increase or decrease)? Who supports you in relation to physical activity
*Step counter*	What do you think about the step counter What does the step counter mean to you What do you think the step counter is going to mean to you in the future
*Closing questions* Makes it possible for the interviewees to raise spontaneous issues, inspired by the previous questions	Is there anything else you would like to tell me about
*Exploratory questions* Makes the interviewees feel important. These questions are used when appropriate throughout the interview	That sounds interesting, please tell me more Can you give me a more detailed description? Please, provide examples

## Appendix 2

**Table 5:** Interview guide to health professionals

*Opening question*	
Who, time and place	Would you please introduce yourself briefly by telling your name, profession and the place of your employment? (Healthcare centre or hospital)
*Research questions*	*Interview questions*
The health professionals experienced of using the pedometer in the interaction with the patients concerning physical activity	Tell about the step counter What is your experience of using the step counter as a working tool to support the patients’ physical activity? What do you think the step counter means to the patients? Tell about the relatives involvement in the patients use of the step counter
*Step counter – own experiences*	Have you used the step counter yourself? What do you think about the step counter?(as a working tool) What does the step counter mean to you Does it influence your interaction with the patient that you have a step counter yourself
*Closing questions* Makes it possible for the interviewees to raise spontaneous issues, inspired by the previous questions	Is there anything else you would like to tell me about
*Exploratory questions* Makes the interviewees feel important. These questions are used when appropriate throughout the interview	That sounds interesting, please tell me more Can you give me a more detailed description? Please, provide examples

## Appendix 3

**Table 6:** Deductive content analysis and resulting themes

Units of meaning			Sub-themes	Themes
Interview: patients	Interview: health professionals	Observations and Documents (notes from the PHR)		
*Autonomy*				
“Well, I don’t do it for the sake of others, only for my own sake. It’s the same about the goal of 10,000 steps, which I might not get to every single day, but then I get more another day” (ID5). “When the weather was nice, I could easily walk the 10,000 steps. But, I would not have walked 10,000 steps on a rainy day [laughs]. I want to decide myself” (ID12). “When the lawn needs mowing, then you feel motivated, not to be active, but to make the lawn look good” (ID8). “I don’t need the pedometer anymore. I now know how many steps the normal working day provide, or my favourite walking trip. If I have been inactive, then I just walk 18 holes at the golf club” (ID5). “The alternative was to exercise at the Healthcare centre, but I am not driving all that way for half an hour of exercise. You could just take a walk in the nature. It’s basically the pedometer that supports your exercise” (ID11).	You have to accept the patient’s choice At the same time you have to make sure that the patient understands the health related problems of their choice, and that the choice are made on the basis of knowledge (Nurse at Healthcare Centre 3).	“In the middle of the living room there was a rowing machine, and during the first observation the patient expressed a wish for a more detailed personal plan for exercises improving strength beside her pedometer goals. She explained that this was because she wanted to exercise on her own” (Field note ID3). “Morten, you are close to the 5000 steps per day, good ☺, are you ready to increase the amount of steps?” (PHRpatientID12).	Individual choice and decision for walking activity	Independence from standardised rehabilitation
“When you have a pedometer, you look at it, how many steps have I walked now? Then we’ve gone for an evening walk. If you can’t see the results of what you do, that is, measuring the steps, then you have no opportunity to adjust” (ID10). “Before [the pedometer] I wasn’t given any marker on how many steps to walk a day. I was just told to walk; now I walk longer distances about 7000 steps 8000 steps on one trip” (ID2).	“After all we use it a lot, I preach; you must reach those 10,000 steps a day, but we do have some citizens that … if they reach 5000 then I think it’s very well done, considering their physical level” (Physiotherapist at Healthcare Centre1). “In worst case scenarios they only walked 2000 steps in a day. You have to be aware of their starting point when you plan their individual activity level” (Physiotherapist at Healthcare Centre4).	“I can see you are getting close to your goal of steps, should we try and raise the number of steps to 10,000?” (PHRpatientID2). “Thanks for a nice talk on the phone today; I am pleased that you are feeling OK. The heart failure makes you ‘short of breath’ and I suggest that you take shorter but more frequent walks (PHRpatientID7). “John experiences leg pain when walking [just a short distance]. We agreed to measure how far he can walk (numbers of steps). After that we will determine goals for daily steps” (PHRpatientID1). ”Thomas wants to loos weight by increasing physical activity through indoor bike riding and 5000 daily steps. Suggested that Thomas divides the walking trip into two. In a month’s time we will evaluate the achieved physical activity PHRpatientID11).	Tailor walking activity	
*Competence*				
“Unfortunately I forgot the pedometer this morning, and I went for a long walk, which unfortunately didn’t get registered” (ID2). “I forgot my pedometer today, but I went for a shopping trip in Aalborg, and I think I walked about 7000 steps all together. I tell you: I was so tiered after that, I slept all evening” (PHR ID1). It’s nice to see that I did actually walk many steps today.” ID1)	“Previously it seemed blurred, whereas now, with this [the pedometer], it is easier to keep track on their activity” (RN at Healthcare Centre3).	Hi Mette [nurse at the hospital], the pedometer is really motivating. I wore it at the gym, I went there with my wife, and it gave me 2 – 3.000 steps at the cross trainer” (PHRpatientID12).	Feedback on walking activity	Conscious awareness of walking activity
“Especially when I think about it, in a way, I’ve got my life back so, if I just sat back, I wouldn’t have understood ‘the message’” (ID3). “Purely for medical reasons, it is all about your health. It is all about health. I try to keep as healthy as possible, and it appears that, exercise makes a difference” (ID10). “Walking is my work. I want to be in a good shape, because it’s good for me” (ID2). “It’s form my own sake, and if some clever people tells me that 10000 steps per day is good for my, the it won’t be any good if I just walk 500 steps (ID 5)	Some of the patients don’t consider walking as a health related issue; they just consider walking as an act to get from one place to another. The pedometer changed that. (Nurse at Healthcare Centre 1)	“Three months has passed by, and you have to live without telerehabilitation technologies. You have reached all your goals; you have lost 13 cm around your waist, and walk a lot of steps. You have said no to any additional rehabilitation sessions at the health care centre” (PHRpatientID11). “A patient had the pedometer placed visibly at the shirt, expressing that it is an appropriate place for him, because it reminds him to walk and makes him aware of activity” (Field note ID11). “Hi, Helle. It’s nice to see that you really focus on exercise and activity, and that you set yourself personal goals. Regarding strength exercise, I have some suggestions, but it is very important to listen to the signs from your body, like pain. You can make sit ups and by doing … etc. etc. If you have any questions don’t hesitate to contact me again. Yours sincerely, Peter Hansen, Physiotherapist, Aalborg University Hospital” (PHRpatientID3)	Knowledge leading to awareness of walking	
*Relatedness*				
“You lose face if the pedometer shows too few steps. I mean, you lose face if you don’t do what they [the health professionals] told you to do” (ID4). It may not show too few steps … it would be embarrassing to wear a pedometer that only shows 200 steps. It has to be more, maybe not in one walk … but if you continue to walk, then the victory comes to you (ID4). Of cause you listen to people [health professionals] who knows what they are talking about (ID1)	“For the patients, it’s the immediate result each evening. It’s like a close surveillance of activity, like a: ‘well done today, Peter’” (Physiotherapist at Healthcare Centre2).	Hi Ib. How are you? Are you using the pedometer every day? There aren’t many steps uploaded to the PHR (PHRpatientID4). Hi Hans. I can see that you have been really active, that’s good☺. Enjoy the lovely weather today, maybe you feel like a long walk on the beach? (PHRpatientID5) Hallo Mette [nurse at the hospital]. As you can see, I don’t walk much. I am extremely affected by the new drug. Immediately after intake, my pulse and blood pressure drops and I need to lie down. (PHRpatientID4) It’s a safe feeling, that the nurse follows your rehabilitation status in the PHR. I haven’t reach my goals yet, but I am determined that I will (PHRpatientID9)	Feeling of being under surveillance, yet supported	Interaction whit others in relation to walking activity
It’s my kids, my kids they are also active, and I want to be active together with them. They have been walking with me; they helped me to get started. And when you have the pedometer, then you look at it, ‘how many steps have I been walking?’, then we have been out for an evening walk. (ID3). I just call my friend and ask her; ‘don’t you need some fresh air?’ It’s like; ‘two for the price of one’ because then we talk and talk, and suddenly, whiteout noticing, we have been walking a long trip (ID6).	His wife bought a pedometer herself, they compared, and talked about how many steps they had reached. I think that it was motivating for them, to see that the spouse feeling good. (Physiotherapist at Healthcare Centre3)	I offered the patient rehabilitation gym at the healthcare centre, but he chose continues to exercise with his wife (PHRpatientID11).	Support from next of kin	
